# Assessing clinical communication skills in physicians: are the skills context specific or generalizable

**DOI:** 10.1186/1472-6920-9-22

**Published:** 2009-05-15

**Authors:** Lubna A Baig, Claudio Violato, Rodney A Crutcher

**Affiliations:** 1 Alberta International Medical Graduate Program, and Community Health Sciences, University of Calgary, Calgary, Canada; 2 Medical Education and Research Unit, Department of Community Health Sciences, Faculty of Medicine, University of Calgary, Calgary, Canada; 3 Department of Family Medicine, Faculty of Medicine, University of Calgary, Calgary, Canada

## Abstract

**Background:**

Communication skills are essential for physicians to practice Medicine. Evidence for the validity and domain specificity of communication skills in physicians is equivocal and requires further research. This research was conducted to adduce evidence for content and context specificity of communication skills and to assess the usefulness of a generic instrument for assessing communication skills in International Medical Graduates (IMGs).

**Methods:**

A psychometric design was used for identifying the reliability and validity of the communication skills instruments used for high-stakes exams for IMG's. Data were collected from 39 IMGs (19 men – 48.7%; 20 women – 51.3%; Mean age = 41 years) assessed at 14 station OSCE and subsequently in supervised clinical practice with several instruments (patient surveys; ITERs; Mini-CEX).

**Results:**

All the instruments had adequate reliability (Cronbach's alpha: .54 – .96). There were significant correlations (r range: 0.37 – 0.70, *p *< .05) of communication skills assessed by examiner with standardized patients, and of mini-CEX with patient surveys, and ITERs. The intra-item reliability across all cases for the 13 items was low (Cronbach's alpha: .20 – .56). The correlations of communication skills within method (e.g., OSCE or clinical practice) were significant but were non-significant between methods (e.g., OSCE and clinical practice).

**Conclusion:**

The results provide evidence of context specificity of communication skills, as well as convergent and criterion-related validity of communication skills. Both in OSCEs and clinical practice, communication checklists need to be case specific, designed for content validity.

## Background

Communication is one of the most important components of physicians' patient management skills and overall competence. Competence in a physician is a composite of clinical skills, interpersonal aspects of patient physician encounter, professionalism and communication skills [1-3]. A good communicator can extract appropriate history from the patient, formulate an appropriate diagnosis, build a strong doctor patient relationship, and can appropriately negotiate management strategy with the patient.

OSCEs have been used extensively to assess communication skills. Measurement errors have been identified for case specificity, candidate-standardized patient (SP) interaction, and case-candidate interaction [4,5]. Although Hodges, Turnbull, Cohen et al. reported a significant difference in the mean score of difficult and easy OSCE stations, they nevertheless concluded that communication skills are bound with content knowledge and are case or context specific [6].

Guiton, Hodgson, Delandshere and Wilkerson found high internal consistency (Cronbach's alpha 0.89 – 0.94) within 7 OSCE stations [4]. The Cronbach's alpha based on intra-item calculation across cases, however, was low. In their Generalizability analysis they found that the highest variance (50%) was contributed by students by case interaction implying that communication skills are case specific [4]. Conversely, Keely, Myers and Dojeiji found that the internal consistency of one 22-minute station in an OSCE to test written communication skills of 36 Internal medicine residents from year 1 through 4, was 0.80. Moreover, they found that it correlated with a breaking bad news verbal communication station (r = 0.37 *p *< 0.01) but not with the thyroid examination station (r = 0.04 ns) [7].

OSCEs have been widely used to assess communication skills in students, residents, and other physicians for licensing and certification. The assessment of communication skills is still plagued with measurement errors related to content specificity, language proficiency, case and student interactions, variability in standardized patients, and assessment of written communication skills [8]. Most studies have, however, found that even with reliable OSCE stations, the intra-item across case reliability is low [4,6,7]. This low intra-item agreement (i.e., low reliability) across cases or stations indicates that communication skills are content specific.

Humphries used confirmatory factor analysis to identify the model best fitting the communication skills assessed through OSCE by SPs and expert examiners on objective structured video examination (OSVE) [9]. He first identified the latent variables as specified by OSVE, SPs and the experts and then did confirmatory factor analysis to identify the best fitting model that could account for the effect of knowledge on future performance of candidates related to communication skills. He could not find a strong relationship between knowledge and performance of communications skills and concluded that better assessment tools need to be developed to assess this complex trait [9].

In the OSCEs used for Clinical Skills Assessment of IMGs in the United States, SPs assess the candidates for communication, and data gathering skills (inclusive of history taking and physical examination), interpersonal skills and English proficiency [8,10]. There is generally high test-retest reliability for all components and low correlations between English proficiency, interpersonal skills, and communication with measures of clinical competence [8]. Conversely, Colliver and colleagues found high correlations between clinical competence and communication skills (also empathy) for specific cases [11,12].

The foregoing and other studies have uncovered mixed evidence for generic and domain specific aspects of communication skills [4,6-8,10,13,14]. Accordingly, further research is needed to investigate the issue of the domain specificity of assessment tools used for assessing communication skills in physicians. The purposes of the present study were to 1) study the psychometric characteristics of an instrument to assess communication used in high stakes OSCEs, and 2) investigate the specificity or generality of communication assessed in OSCEs vis à vis communication assessed in clinical practice.

## Methods

### Study Design

A psychometric study design was employed to investigate the reliability and validity of communications skills instrument used for high-stakes examination.

### Context of the Study

The Western Alliance for Assessment of International Physicians (WAAIP) project was created to develop and field-test an assessment process to determine the practice readiness of selected international medical graduates (IMG) registrants identified by College Registrars in Western and Northern Canada. The intent was to facilitate IMG integration into clinical practice while maintaining Canadian clinical standards. [15] Four provinces (Alberta, Manitoba, Saskatchewan, and British Columbia) and the Northwestern Territories nominated 39 physicians for practice ready assessment. We anticipated that if successful they could apply for a restricted license to practice medicine in the respective province or territory. The study was approved by the University of Calgary Conjoint Health Research Ethics Board.

Assessment occurred in two parts: 1) Step A, a 150 item multiple choice questions exam to test declarative knowledge followed by a 14 station objective structured clinical exam utilizing standardized patients for testing clinical and communication skills, and 2) Step B, direct assessments and evaluations of the IMGs in a three month supervised clinical practice experience. During supervised clinical practice, several direct observation instruments as well as patient surveys for assessing varied competencies including communication were employed.

Communication skills were assessed both by the physician examiner and the standardized patients (SPs) for each OSCE station. The same instrument was used by the physician assessor and SP. The communication skills instrument had 13 items and rated from 1–5 (1 = strongly disagree, 3 = neutral, 5 = strongly agree). In Step B the top performing 25 candidates were selected for supervised clinical practice of 12 weeks in their respective provinces. They were assessed through the instruments employing direct assessments in supervised clinical practice employing Physician Achievement Review (PAR) [16], Mini-CEX [17], and In Training Examination Reports (ITERs). The Mini-CEX is a 9-point scale and PAR is 5 point scale (1 = strongly disagree, 3 = neutral, 5 = strongly agree), and communication items on ITERs are a 3-point scale.

### Data-analysis

SPSS Version 14 was used to calculate the descriptive statistics, factor analysis and Cronbach's alpha for inter- and intra-item across station reliabilities. OSCE scores of 39 candidates were used to calculate inter- and intra-item across stations reliabilities. Scores of 24 successful candidates for communication items on OSCEs communications checklist, PAR, ITERs and Mini-CEX were used for developing the correlation matrix and factor analysis. Generalizability analyses were conducted for the communication checklist in a nested design (SPs within cases).

### Subjects

A total of 39 physicians (19 men – 48.7%; 20 women – 51.3%) who had graduated from a medical school included in the World Health Organization's directory of medical institutions and had a medical degree verified by the Educational Commission for Foreign Medical Graduates International Credentials Services participated. Each candidate had met the minimum required standards on the *Test of English as Foreign Language *(TOEFL) and had passed the Medical Council of Canada Evaluating Examination (MCCEE).

The mean age was 41 years (SD = 6.5; range: 29 – 55 years). The mean postgraduate clinical experience was 16.23 years (SD = 6.9; range: 4 – 30 years). Fifteen (38.4%) physicians originated from Asia, six (15.4%) from Eastern Europe, fifteen (38.4%) from the Middle East, and three (7.8%) categorized as 'other'.

## Results

Twenty-five of 39 IMGs (with equal success rates between males and females) passed Step A and 24 (1 withdrew) moved to Step B and were assessed during supervised clinical practice. Out of these 24 IMG's, based on the assessments during supervised clinical practice 16 passed Step B and subsequently obtained a restricted license to practice in their respective provinces.

The Cronbach alpha reliabilities of the communication instrument used in the OSCE stations by the SPs and by the physician assessors are summarized in Table 1, as are the descriptive statistics. These alphas ranged from .54 to .94. In general these alpha coefficients are quite high indicating substantial internal consistency of the instrument. The generalizability analysis for communication checklist scores across cases with SP's nested within cases yielded Ep^2 ^= .62. The percent of variance attributable to participants was 5.4, cases 4.6, participants by cases 45.0 and cases by raters' (raters' were assigned to cases and not nested) was 45.0.

**Table 1:** Descriptive Statistics and Reliability for the OSCEs Standardized Patients and Physician Examiners Communication Checklist

	**Mean ± SD**		**Cronbach's Alpha**	
	
**The OSCE Stations**	**SP**^**†**^	**PE**^**††**^	**SP**	**PE**
**1. Chest pain**	3.91 ± 0.16	3.64 ± 0.34	0.89	0.87

**2. Fatigue**	3.75 ± 0.39	3.60 ± 0.43	0.94	0.93

**3. Stomach flu**	3.96 ± 0.44	3.89 ± 0.42	0.90	0.87

**4. Headaches & loss of appetite**	4.04 ± 0.25	3.81 ± 0.26	0.88	0.82

**5. Severe headache**	4.26 ± 0.53	3.76 ± 0.36	0.80	0.91

**6. Risk management**	3.62 ± 0.61	3.75 ± 0.26	0.58	0.89

**7. Problems urinating**	4.42 ± 0.16	3.96 ± 0.24	0.91	0.89

**8. Pre-operative counselling for appendicitis**	3.54 ± 0.87	3.51 ± 0.80	0.78	0.79

**9. Severe leg pain**	4.18 ± 0.29	3.81 ± 0.32	0.93	0.79

**10. Breaking bad news**	3.66 ± 0.34	3.57 ± 0.32	0.90	0.87

**11. Vomiting blood**	3.39 ± 0.59	3.39 ± 0.42	0.93	0.78

**12. Loss of sensation in thumb & fingers**	3.30 ± 0.46	3.47 ± 0.44	0.87	0.88

**13. Shortness of breath**	3.40 ± 0.63	3.41 ± 0.34	0.54	0.86

**14. Fever after cholecystectomy**	3.71 ± 0.48	3.39 ± 0.40	0.88	0.93

^† ^Standardized patient

^†† ^Physician examiner

The intra-item reliabilities across cases are summarized in Table 2 for both the physician examiner and the standardized patient (range: .13 to .56). Unlike the instrument alphas, these coefficients are quite low indicating poor intra-item agreement across cases.

**Table 2:** Intra Item across Case Reliability and Mean of Items on the Communication Checklist

**Items on the Communication Checklist (n = 14 OSCE stations)**	**Mean**		**Cronbach's alpha**	
	
	**PE**	**SP**	**PE**	**SP**
1. The doctor wanted to understand how patients saw things	3.71	3.94	0.42	0.33

2. The doctor usually sensed what the patient was feeling	3.71	3.92	0.50	0.52

3. The doctor just took no notice of some things that the patient felt or thought^†^	3.72	3.60	0.23	026

4. The doctor's response to the patient was so fixed that the patient didn't really get through to him/her^†^	3.73	3.53	0.41	0.13

5. The doctor treated patient with respect and courtesy	4.29	4.40	0.25	0.20

6. The patient was able to explain his/her problem to the doctor as fully as needed	3.60	3.94	0.26	0.23

7. The doctor explained things to the patent so that they know what may be the matter with them	3.59	3.73	0.29	0.33

8. The doctor explained what treatment tests or other follow up is going to happen	3.49	3.65	0.32	0.34

9. The doctor gave the patient opportunity to express his/her feelings or ideas in planning treatment tests or follow-up	3.17	3.25	0.51	0.40

10. The doctor gave the patient opportunity to ask questions	3.74	3.61	0.55	0.49

11. The doctor used understandable and non-technical language	3.88	3.98	0.53	0.35

12. The doctor was careful and thorough	3.59	4.00	0.56	0.45

13. The patient feels satisfied with the medical care that he/she received	3.37	3.82	0.53	0.45

^†^The scale was reversed during analysis: 5 = strongly disagree to 1 = strongly agree

The descriptive statistics on the communication items for the instruments used for assessing IMG's during supervised clinical practice are given in Table 3. Most candidates did well on the communication as can be seen from the high mean and small standard deviation of the scores. (Table 3)

**Table 3:** Descriptive Statistics and Reliability for the communication items on ITER's Mini-CEX and PAR instruments

**Instruments**	**Mean+ SD**	**Min**	**Max**	**Cronbach's Alpha**
**1. ITER's communication**^**†**^	2.88 ± 0.17	2.48	3.00	0.85

**2. PAR Co-worker communication**	4.12 ± 0.46	3.5	4.81	0.86

**3. PAR Patient communication**	4.44 ± 0.26	3.9	4.95	0.92

**4. Mini-CEX Interviewing skills**	6.73 ± 0.85	4.86	8.56	0.54

^†^Communication skills was a 3-point scale with 1 being the lowest and 3 being the highest

The communication scales (alphas range: .51 to .96) from the various measures were intercorrelated (Pearson's r) – the results are summarized in Table 4. There were significant correlations (*p *< .01) between OSCE physician assessors with SPs, and of mini-CEX with PAR patient communication, and ITER communication. The two PAR instruments had moderately significant correlations (*p *< .05) with each other and ITERs (Table 4).

**Table 4:** Correlation Matrix of Communication Skills in OSCEs and Supervised Clinical Practice

	**Mini-CEX Communication**^**1**^	**OSCE-SP^**2 **^Communication**	**OSCE- Physician^**3 **^Communication**	**PAR Co-worker Communication**	**PAR Patient Communication**
**OSCE-SP Communication**	0.30				

**OSCE- Physician Communication**	0.21	0.70**			

**PAR Co-worker Communication**	0.43	0.27	0.11		

**PAR Patient Communication**	0.61**	0.16	-0.03	0.43*	

**ITER Communication**	0.50*	0.10	0.10	0.24	0.37*

* *p *< 0.05 ** *p *< 0.01

^1^Score of Medical Interviewing skills on the Mini-CEX was used as a measure of communication skills (Dr. Patient communication)

^2 ^Score on Communication Checklist used by the standardized patient

^3 ^Score on Communication Checklist used by the physician

Principal component analysis was done with varimax rotation, which converged in 3 iterations. The factor analysis of all the instruments together yielded a two-factor solution accounting for 67% of the total variance in the scores of communication skills (Table 5).

**Table 5:** Rotated factor analysis with Kaiser Normalization of communication skill using communication scores on OSCE and the instruments used during supervised clinical practice (PAR, ITER's and Mini-CEX)

	**Cronbach's Alpha**	Components	
		
		**Application of Communication Skills**	**Knowledge of Communication Skills**
**Overall Examiner Communication**	0.87	-	0.97

**Overall SP Communication**	0.88	-	0.93

**PAR Co-worker Communications**	0.86	0.80	-

**PAR Patient Communication**	0.92	0.80	-

**ITER Communications**	0.85	0.44	-

**Mini-CEX Medical Interviewing Skills**	0.54	0.81	-

## Discussion

The main findings of the present study are: 1) The instruments used for assessing communication skills during supervised practice had good internal consistency reliability; 2) The communication skills instrument used for OSCEs had good reliability within each OSCE station; 3) For the 13 items on the checklist the intra-item reliability across all cases was very low. This means that the candidates' performance varied substantially for all items of communication skills; 4) While the generalizability coefficient indicated adequate data stability overall, the high variance attributed to cases by raters means that error was introduced by raters for same items on different cases; 5) There were significant correlations for communication assessment within clinical practice, but not between clinical practice and OSCE assessments; 6) The factor analysis of all instruments combined yielded a 2-factor solution separating performance from assessment of knowledge application during OSCE.

The lack of correlations between the communication measures from the OSCE (but significant correlations by SP and physician assessors) and clinical practice suggests method specificity of the measures. The correlations of communication measures within clinical practice (PAR patients, PAR co-workers, mini-CEX, and ITERs) further support the method specificity of communication assessments. The OSCE is a standardized, comparatively structured task where the candidates know that it is an examination. The assessment during clinical practice was naturalistic and much less structured than the OSCEs although the mini-CEX might be considered 'semi structured'. The correlations within the naturalistic setting provide evidence of convergent and criterion-related validity for assessing communications. Similarly, the correlations within the OSCE measures (SPs and physician assessors) also provide evidence of convergent and criterion-related validity. The lack of between method correlations provides evidence of the context specificity of communications.

The context specificity is supported by the low intra-item correlations (alpha) across OSCE stations. So even though the method was consistent (OSCE stations), the same item (e.g., 'the doctor treated patient with respect and courtesy') were not rated consistently across cases for the same candidate. That is, the same candidate may have explained what the problem was to the SP very well for the chest pain (Station 1) but not for fever after cholecystectomy (Station 14). The high internal consistencies (alphas) provide further evidence that the items are inconsistent across stations because of context specificity. This context specificity is further supported by high variance attributed to cases by raters in the generalizability analysis.

Our foregoing results are in concordance with previous findings about the context and case specificity of communication skills [4,6,8,10]. We are in agreement with Hodges, that communication skills are domain specific. Accordingly, communication checklists should be specific and tailored for each case as one checklist for all cases appears inappropriate. An item such as "the doctor used understandable and non-technical language" may apply differently with a technical case (e.g., Pre-operative counselling for appendicitis) compared to one that is not as technical but much more emotionally charged (e.g., Breaking bad news).

The 2-factor solution of all the instruments together further disconnects the OSCEs from the assessment instruments used during supervised training. All the instruments used at Step B loaded on to the same factor with no split loadings from OSCEs checklist. This could either be due to method effect or that OSCEs are testing knowledge application in a contrived setting and may not necessarily predict performance in a real doctor patient encounter. It could also be due to the fact that cases in OSCEs (with SPs) are different from real cases and that communication skills are content and case specific. These results are in conformity with earlier studies that have shown that communication during a doctor patient encounter is influenced by many factors ranging from knowledge of physician to interpersonal, and other non-cognitive attributes [9,11-14].

If we assume that OSCEs test the knowledge of communication skills and Mini-CEX, PAR and ITERs, test application of knowledge then the results are in conformity with the study by Humphries [9]. The moderate to strong relationships between communication skills instruments used during supervised training could either be due to similar testing situations or method specificity.

A limitation of the present study is the relatively small sample size and its composition. The correlations that we found may be unstable because of the modest sample. As well, the sample consisted of IMGs seeking licensure to practice medicine in Canada. Future research should focus on replicating and extending our findings with other participants including local medical graduates (and thus native language speakers), residents and physicians in independent practice.

## Conclusion

The results of the present study provide evidence of content and domain specificity of communication skills. This means that communication checklists should be specific and tailored for cases; a generic instrument may not be useful for all cases. Notwithstanding the limitations of the present study, our results are in concordance with other findings and underscore the need for refinement in the assessment procedures for communication skills that is currently done.

## Competing interests

The authors declare that they have no competing interests.

## Authors' contributions

LB Identified the research question and lead with the data analysis, did the first draft of the paper and subsequent revisions.

CV contributed to the data analysis and did major revisions of the manuscript.

RC was the co-leader of WAAIP, and contributed to the conceptualization and operationalization of all phase of the project, and contributed to development and revision of the manuscript. All authors read and approved the final manuscript.

## Pre-publication history

The pre-publication history for this paper can be accessed here:


